# Protocols for Reporting Speech Outcomes following Palatoplasty or Velopharyngeal Surgery: A Literature Review

**DOI:** 10.1097/GOX.0000000000002151

**Published:** 2019-02-08

**Authors:** Ann W. Kummer, Hedieh Hashemi Hosseinabad, Erin Redle, Stacey Clark

**Affiliations:** From the * Division of the Speech-Language Pathology, Cincinnati Children’s Hospital Medical Center, Cincinnati, Ohio; † Department of Pediatrics, University of Cincinnati College of Medicine, Cincinnati, Ohio; ‡ Department of Otolaryngology, University of Cincinnati College of Medicine, Cincinnati, Ohio; § Department of Communication Sciences and Disorders, College of Allied Health Sciences, University of Cincinnati, Ohio; ¶ Department of Communication Sciences and Disorders, Eastern Washington University, Spokane, Wash.; ‖ Department of Otolaryngology, Dell Children’s Medical Center at the University of Texas, Austin, Tex.

## Abstract

**Background:**

To determine best practices, surgeons who perform cleft palate surgery or surgery for velopharyngeal insufficiency need to be able to compare their outcomes in normalizing the velopharyngeal valve.

**Methods:**

We conducted a comprehensive review of articles that reported speech/resonance outcomes following palatoplasty or surgery for velopharyngeal insufficiency. We analyzed protocols that were used and how the results were reported. We found 170 articles, published between 1990 and 2014, that met our inclusion criteria.

**Results:**

Most studies (66%) had a sample size of <50 subjects, were retrospective (67%), were not blinded (83%), and did not report the use of reliability (68%). Most studies included 1 evaluator (27%) or 2 evaluators (30%). Only 80% of the articles specified that at least one speech pathologist was an evaluator. Most articles (56%) did not specify the speech samples used, and 65% used an informal test or did not specify the type of test used. Most studies used a perceptual rating scale for articulation (75%) and resonance (83%). Only 39% of the studies included an evaluation of velopharyngeal function. Finally, objective measures were used in only 28% of the studies (9% used aerodynamic measures and 19% used nasometry).

**Conclusions:**

Because these articles showed significant variability in how speech/resonance is evaluated and how the outcomes are reported, it is virtually impossible to compare results to determine best surgical procedures. Suggestions are given to standardize outcome measures to improve comparability of data.

## INTRODUCTION

The primary purpose of cleft palate repair (palatoplasty) is to normalize palatal structure and velopharyngeal function so that the patient will be able to develop normal speech. Despite the palate repair, about 20%–30% of children with history of cleft palate will have velopharyngeal insufficiency (VPI).^1^ Correction of VPI requires a secondary procedure (ie, pharyngeal flap, sphincter pharyngoplasty, Furlow Z-plasty) and sometimes secondary surgery revisions.

There are several surgical procedures designed to repair a cleft palate and also several procedures to correct VPI. Unfortunately, there is disagreement about which palatoplasty procedure has the lowest rate of VPI and which pharyngoplasty procedure is the best for correction of VPI.^2^ For example, in 2012, Kummer et al.^3^ reported the results of a survey of cleft palate professionals who were asked which secondary surgical procedure is done most often in their center. More than half of the respondents (52.9%) reported that the pharyngeal flap was the most commonly used procedure at their center. Sphincter pharyngoplasty was reported as the preferred procedure by 27.5% of respondents, and Furlow Z-plasty was reported as the most common procedure by 19.6% of respondents.

Ideally, the selection of a surgical procedure should be done based on evidence of the efficacy of that procedure in comparison to other procedures, rather than on surgeon’s previous training or opinion. The only way to determine which surgical procedures are most effective (and in which circumstances) is to compare clinical outcome data between the procedures. If there is inconsistency in the method of evaluating and reporting surgical results and even in the criteria for determining a successful speech outcome, individual studies cannot be compared with each other and surgical selection will continue to be based on the surgeon’s preference alone.

The aim of this literature review, therefore, was to determine the current methods and typical protocols used for assessing and reporting speech/resonance outcomes as a result of cleft palate and VPI surgery. (It should be noted that this literature review was not done to evaluate the results of these studies, to judge the quality of the research, or to examine the strength of the evidence.) The ultimate goal of this research is to develop consistent and appropriate assessment and reporting protocols so that intercenter comparisons can be made. Once like comparisons of data can be made, it will be possible to determine which surgical procedures are truly most effective. This will ultimately improve overall speech outcomes, decrease the need for revision surgeries, and decrease the burden of care on children and their families.

## METHODS

Two literature searches of 4 electronic databases [ie, PubMed (Medline), Web of Science, Cumulative Index to Nursing, Allied Health Literature, and Cochrane] were performed to identify all published studies that reported speech outcomes following palatoplasty and/or VPI surgery published over a 25-year period (from January 1, 1990 to December 31, 2014). Search terms included: cleft palate, surgical outcomes, speech outcomes, primary palate surgery, palatoplasty, pharyngoplasty, pharyngeal flap, sphincter pharyngoplasty, Furlow palatoplasty, and velopharyngeal insufficiency. The concept map and search query development were used as search strategies.

Inclusion criteria were as follows: published between 1990 and 2014, peer-reviewed research publication, reported speech outcomes following primary palate and/or secondary VPI surgeries, and published in English. Exclusion criteria included articles reporting speech outcomes following maxillary advancement.

A total of 278 articles were initially identified through the database searches. The abstract of each article was then read by 2 reviewers to determine if the article met the full inclusion criteria. As a result of this initial review, 90 articles were excluded. The full texts of the remaining 188 articles were obtained from the Health Sciences Library at the University of Cincinnati and from the Pratt Library at Cincinnati Children’s. After the reviews, another 18 articles were excluded for following reasons: not peer-reviewed (n = 3), reported speech outcomes were following orthognathic surgery (n = 8), reported speech outcomes were following prosthetic management (n = 3), and the speech outcomes were not following surgery (n = 4).

After exclusions, 170 articles remained and were included in this literature review. These articles came from 38 different journals in 5 main categories: 88 articles (52%) were in journals of *Plastic and Reconstructive Surgery*, 47 articles (28%) were in the *Cleft Palate-Craniofacial Journal*, 24 articles were in journals related to otolaryngology (14%), 5 articles were in journals related to speech-language pathology (3%), and the remaining 6 articles (3%) were found in other miscellaneous journals.

The years of publications were categorized into 5-year segments for representation of the data. The number of articles by period were as follows: 10% were from 1990 to 1994, 20% were from 1995 to 1999, 18% were from 2000 to 2004, 25% were from 2005 to 2009, and 27% (the largest amount) were from 2010 to 2014.

Of these articles, 57% reported speech outcomes following primary palatal surgery, 35% reported speech outcomes after secondary surgery for VPI, and 8% reported outcomes following both types of surgery.

The articles were divided up between a team of 4 raters (3 speech-language pathologists and 1 otolaryngologist) for an in-depth review. Using a data extraction form, the raters coded the articles based on various aspects of the study design; assessment protocol; and methods of rating characteristics of speech, resonance, and velopharyngeal function.

To ensure reliability, 10% of the articles were reviewed and coded a second time by another rater. There was 94% agreement among the raters for the second review. All data were pooled into groups within each category, and the frequency of occurrence for each group within each category was calculated.

## RESULTS

### Distribution by Number of Subjects

The number of subjects varied greatly in the studies reviewed. There were fewer than 20 subjects in 19% of the articles, between 21 and 50 subjects in 37% of the articles, between 51 and 99 subjects in 20% of the articles, and between 100 and 199 subjects in 12% of the articles. Only 12% of the articles had a large sample size of ≥200 subjects.

### Distribution by Study Design (Retrospective Versus Prospective)

Of the studies reviewed, 67% were retrospective, 26% were prospective, 2% used both designs, and 5% were unclear as to the design.

### Distribution by Blindness of Speech Evaluations

In most studies (83%), the evaluators were not blinded or blindness was not specified. Only 17% of the studies were blinded.

### Distribution by Reliability Measurement

**Fig. 1.**
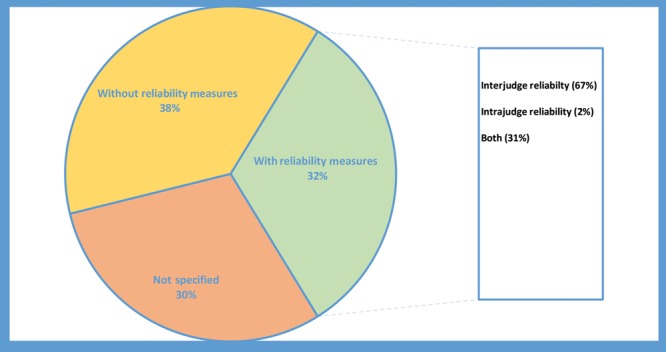
Reliability measures in reviewed articles (n = 170).

In 68% of the studies, reliability measures were not included (Fig. 1). Of the 32% that included reliability measures, interjudge reliability was assessed in 67% of the articles, intrajudge reliability was assessed in 2%, both inter- and intrajudge reliability were assessed in 31% of articles.

### Distribution by Number of Speech Evaluators

In 27% of the articles, there was 1 evaluator; in 30%, there were 2 evaluators; and in 15%, there were ≥3 evaluators. In 28% of the articles, the number of evaluators was not specified.

### Distribution by Type of Speech Evaluators

The speech outcomes were determined by a speech-language pathologist in 80% of the articles. In the remaining 20%, the evaluator(s) was unclear.

### Distribution by Type of Speech Samples

**Fig. 2.**
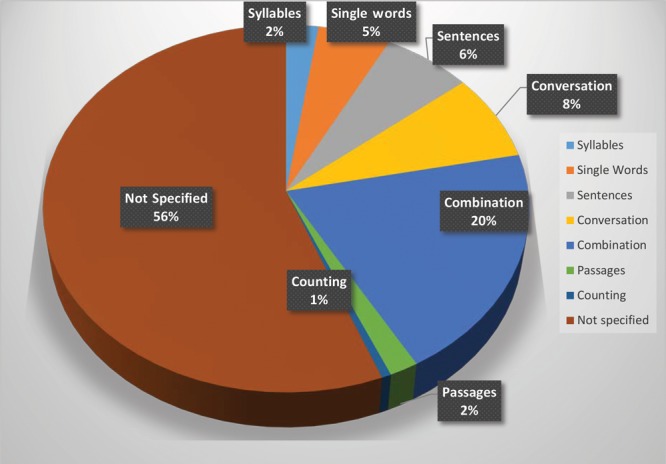
Speech samples for perceptual judgment in reviewed articles (n = 170).

In most articles (20%), the speech samples used for perceptual assessments included a combination of words, sentences, and conversational speech (Fig. 2). However, 56% of the articles did not report the specific speech samples used.

### Distribution by Speech Assessment Protocols

**Fig. 3.**
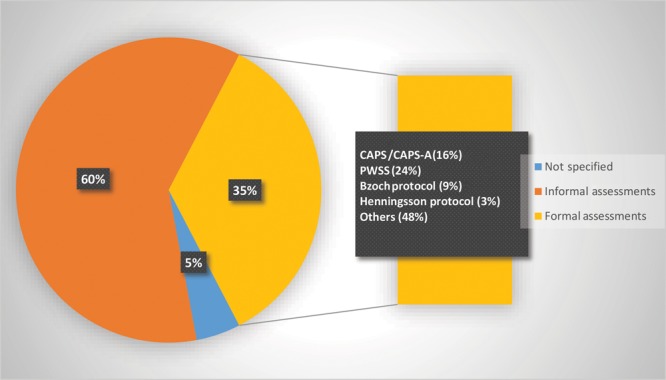
Type of speech assessment protocols in reviewed articles (n = 170). CAPS, Cleft Audit Protocol for Speech; CAPS-A, Cleft Audit Protocol for Speech-Augmented; and PWSS, Pittsburgh Weighted Screening Speech.

Formal assessment protocols were used in only 35% of the studies (Fig. 3). The most common protocols that were used are listed in the figure. The rest of the articles (65%) reported either using an informal protocol or did not specify what was used for the speech assessment.

### Distribution by Speech Parameters Evaluated

Table 1 lists speech parameters that were assessed in the evaluations. Resonance was the most frequently reported in the articles (84%). Surprisingly, articulation was second in frequency (60%) and reported in more articles than nasal emission (56%), which was the third. Velopharyngeal function was only reported in 39% of the studies.

**Table 1.**
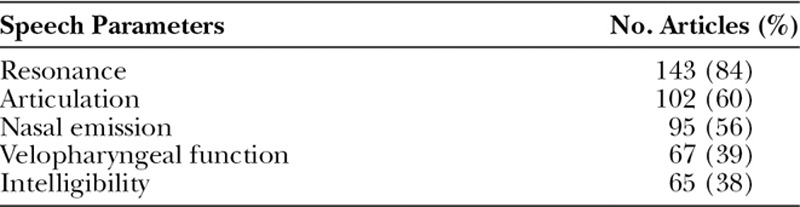
Speech Parameters Evaluated in Reviewed Articles by Order of Frequency (n = 170 in Each Parameter)

### Distribution by Methods of Evaluating Articulation

Of the 102 articles that reported articulation results, 76% used a type of interval scale. The use of a 5-point interval scale was the most common. In 24% of these articles, another method of assessing articulation was used or the method was not clearly explained.

### Distribution by Methods of Evaluating Resonance

As with articulation, most articles (84%) assessed resonance using an interval scale (Table 2). In this case, a 4-point interval scale was the most common. In 17%, the method for assessment of resonance was not specified.

**Table 2.**
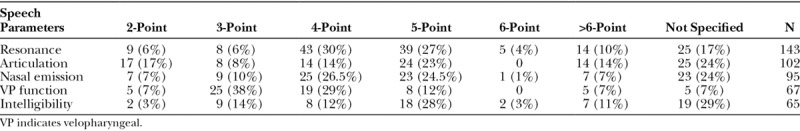
Use of Interval Scaling in Reviewed Articles for Each Speech Parameter Evaluated

### Distribution by Methods of Evaluating Velopharyngeal Function

Only 39% of the studies included an evaluation of velopharyngeal function. In this area of assessment, interval scales were also commonly used, primarily a 3-point scale. It should be noted that 7% of the articles did not specify the method of judgment.

### Distribution by Instrumental Procedures

**Fig. 4.**
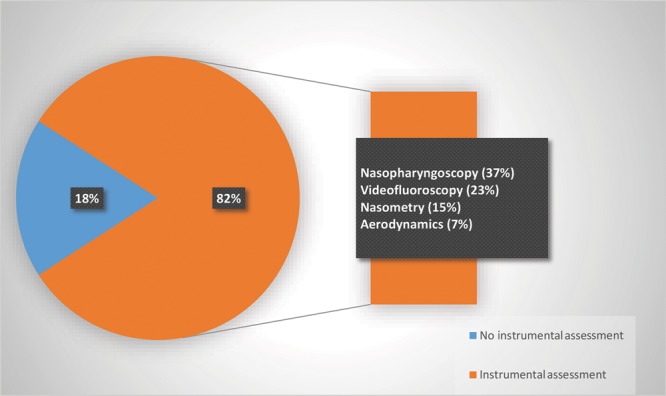
Type and percentage of instrumental assessments in reviewed articles (n = 170).

In 82% of the studies (139), an instrumental procedure was reported as part of the assessment protocol (Fig. 4). Direct instrumental procedures (eg, nasopharyngoscopy and videofluoroscopy) were used by 60% of the articles, whereas indirect, objective measures (eg, nasometry and aerodynamic measures) were reported in only 22% of the articles. Nasopharyngoscopy was reported in 37% of the articles and therefore, was the most commonly used procedure.

## DISCUSSION

### Results and Observations

If the primary goal of palate repair and secondary surgery for VPI is to obtain *normal* velopharyngeal function, surgeons need to know if and how often they are able to achieve that goal. They also need to know how their results compare with those of other surgeons who use different techniques.

To determine how the speech results of cleft palate and VPI surgery are currently reported, a literature review was done on 170 articles over a 25-year period. The results of this review showed significant variation in assessment protocols and methods for reporting speech outcomes.

Most of the studies had a small sample size (<50 subjects), were retrospective, were not blinded, and had no reliability measures. Most had 1 or 2 raters, and about 20% did not include an assessment by a speech-language pathologist. Assessment procedures varied in the type and extent of the speech samples, in the evaluation protocols, and even in the characteristics that were evaluated. Most used an interval scale for rating the severity of speech characteristics, but the scales ranged from 2 to >6 points. Only 38% of studies used available objective measures. Overall, there was significant variability as was found in previous studies,^4,5^ and this does not seem to have been improved in the last few decades.

Of particular interest is the fact that the ultimate definition of success varied greatly among studies. Most studies used subjective descriptors to describe the outcomes of patients in the success group. These descriptors included: *acceptable*, *adequate*, *improved*, *satisfactory*, *good*, *favorable*, *stable*, *intelligible*, and *within normal limits* to describe postoperative speech. The most common criterion for success after palatal or velopharyngeal surgery was simply “acceptable speech,” which is hard to define and subject to the interpretation of the evaluator. Few studies used *normal speech* as the criterion for success. Some studies included patients with mild VPI or postoperative hyponasality and/or sleep apnea in their success category, whereas others did not.

### History of Cleft Outcomes Research

In 2002, the World Health Organization released a report that emphasized a strong need for collaborative, intercenter outcomes research.^6–8^ The Eurocleft studies published in 1992 provided a strong model for successful intercenter comparison research of clinical outcomes of care of patients with cleft lip/palate.^9–13^

Although there have been several intercenter studies on cleft care outcomes in Europe, centers in North America have lagged behind their European colleagues in this type of research. As such, North America has generated very little useful information to date about cleft outcomes that would contribute to the establishment of sound evidence-based practice.^14^

To promote the standardization of measurements for intercenter comparisons, the American Cleft Palate-Craniofacial Association formed “Americleft” in 2006, following the Eurocleft model.^15^ The aim of the Americleft was to standardize reporting of outcomes so that intercenter comparisons could be made. The Americleft Speech Project was started in 2009 with the purpose of developing a methodology for collecting speech data for intercenter comparisons. In cleft palate speech assessment, there has been a lot of debate in the past about what should be assessed and how it should be assessed.^4,16^ Therefore, after much consideration, the group chose a methodology that was based on the Scandcleft procedures,^17^ aspects of the Universal Parameters for Reporting Speech Outcome in Individuals with Cleft Palate,^18^ and a modified version of the Cleft Audit Protocol for Speech–Augmented (CAPS-A), which was developed in the United Kingdom.^19,20^

Both the CAPS-A tool and the modified version used in the Americleft Speech Project rely heavily on listener perceptual judgments of various speech parameters, including hypernasality and nasal emission. These parameters are rated using an equal appearing interval scale. Because inter-rater reliability is key to the ability to make valid comparisons, listener training sessions have been held at several American Cleft Palate-Craniofacial Association meetings. In a recent study of the effectiveness of listener training on inter-rater reliability, it was found that improvements in inter-rater reliability could be obtained following a program of systematic training, but improvement was not uniform across all speech parameters.^21^

### Issues with Speech Outcomes Reporting and Comparisons

In reviewing the articles in this study, there seem to be several barriers to developing an easy, valid, and reliable method to report outcomes for intercenter comparisons. They are as follows:

- *Rating levels of severity*: Inter-rater and intrarater reliability depends greatly on the examiner’s experience. However, even among experienced listeners, acceptable interjudge reliability is difficult to obtain. Although listener training may improve reliability, training is costly, time-consuming, and limited to those who can attend the training. In addition, when there is VPI, the size of the opening and thus the severity level varies with utterance length, loudness, phonemic complexity, and level of effort and fatigue. Therefore, ratings of severity are inherently unreliable. Even if the severity ratings had perfect intra- and interjudge reliability, these perceptual ratings of severity have little to no impact on surgical management decisions.
- *Focus on speech samples rather than speech production*: A speech sample is just that—a sample of typical conversational speech. The particular words and sentences used are not important. It is important, however, to have many samples of all speech sounds in the language, at least at the sentence, rather than single word, level.
- *Confounding effects of speech production versus velopharyngeal function*: Velopharyngeal function depends on normal structure and physiology. In contrast, speech production is a learned, behavioral activity. Therefore, if there is an attempt to compare the surgical outcomes on the function of the velopharyngeal valve, speech articulation needs to be separated from velopharyngeal function in the reporting of outcomes. This is particularly important if the child has developed and maintained compensatory productions due to preoperative VPI. These productions are typically produced in the pharynx, and therefore there will still be nasal emission on these particular sounds postoperatively. Testing velopharyngeal function on sounds with normal placement would be important to separate out the effect of velopharyngeal function versus the learned compensatory articulation production.
- *Lack of objective measures*: Comparison of surgical outcomes has been hampered by the lack of the use of instrumentation to obtain objective and comparable data. Neither nasopharyngoscopy nor videofluoroscopy are adequate for determining outcomes, as they are both subjective and also invasive. Nasometry (PENTAX Medical, Montvale, NJ) is an objective measure of the acoustic correlates of velopharyngeal function, yet few studies in the literature report nasometry data as part of their outcome measures. This may be due to the lack of access to instrumentation, or it may be because there is not always a good correlation between the perceived severity of the speech distortion and the nasalance score due to the fact that the nasalance score measures both nasal emission and hypernasality. If the velopharyngeal gap is made smaller with surgical intervention (but not completely closed), hypernasality will decrease, but the acoustics of nasal emission will increase due to the increased audibility of airflow going through a small opening. Although nasalance scores cannot be used to compare the *severity* of speech or VPI among subjects, these scores are very consistent when there is normal velopharyngeal closure during speech.

Because the goal of cleft palate surgery and surgery for VPI is normal velopharyngeal function, perhaps outcome studies should focus on the percentage of patients at each center who have normal velopharyngeal function postoperatively as judged by a speech pathologist and maybe even the family. Inter-rater reliability of judgments of normal resonance and no audible nasal emission should be very high. The perceptual assessment could be augmented by an objective analysis of velopharyngeal function through nasometry, which excludes passages (typically sibilants) if the child uses compensatory productions in the pharynx for these sounds. Perhaps comparing percentage of patients with normal velopharyngeal function postoperatively could provide evidence for which surgical procedures are most effective.

## CONCLUSIONS

The results of this study demonstrate that there is still significant variability in the literature regarding the way outcomes of surgery for velopharyngeal function are reported. A particular issue is that the definition of success is not consistent and is rarely based on normal velopharyngeal function, and thus normal resonance and no nasal emission.

Because the goal of surgery is normal velopharyngeal function, it is suggested that centers compare with each other their percentage of patients with normal velopharyngeal function as judged both perceptually and through objective measures.

## References

[1] Kummer AW Cleft Palate and Craniofacial Anomalies: Effects on Speech and Resonance. 2014 3rd ed Clifton Park, NY: Cengage Learning.

[2] Sie KC Chen EY Management of velopharyngeal insufficiency: development of a protocol and modifications of sphincter pharyngoplasty. Facial Plast Surg. 2007;23:128–139. 17516340 10.1055/s-2007-979282

[3] Kummer AW Clark SL Redle EE Current practice in assessing and reporting speech outcomes of cleft palate and velopharyngeal surgery: a survey of cleft palate/craniofacial professionals. Cleft Palate Craniofac J. 2012;49:146–152. 21501067 10.1597/10-285

[4] Lohmander A Olsson M Methodology for perceptual assessment of speech in patients with cleft palate: a critical review of the literature. Cleft Palate Craniofac J. 2004;41:64–70. 14697067 10.1597/02-136

[5] Roberts CT Semb G Shaw WC Strategies for the advancement of surgical methods in cleft lip and palate. Cleft Palate Craniofac J. 1991;28:141–149. 1829965 10.1597/1545-1569_1991_028_0141_sftaos_2.3.co_2

[6] Shaw DW Global strategies to reduce the health care burden of craniofacial anomalies: report of WHO meetings on international collaborative research on craniofacial anomalies. Cleft Palate Craniofac J. 2004; 41:238–243. 15151440 10.1597/03-214.1

[7] World Health Organization (WHO). Global Strategies to Reduce the Health-Care Burden of Craniofacial Anomalies. Report of a WHO Meeting on International Collaborative Research on Craniofacial Anomalies. 2002 Geneva, Switzerland: World Health Organization.

[8] World Health Organization (WHO). Addressing the Global Challenges of Craniofacial Anomalies: Report of a WHO Meeting on International Collaborative Research on Craniofacial Anomalies. 2004 Geneva, Switzerland: World Health Organization.

[9] Asher-McDade C Brattström V Dahl E A six-center international study of treatment outcome in patients with clefts of the lip and palate: part 4. Assessment of nasolabial appearance. Cleft Palate Craniofac J. 1992;29:409–412. 1472518 10.1597/1545-1569_1992_029_0409_asciso_2.3.co_2

[10] Mars M Asher-McDade C Brattström V A six-center international study of treatment outcome in patients with clefts of the lip and palate: part 3. Dental arch relationships. Cleft Palate Craniofac J. 1992;29:405–408. 1472517 10.1597/1545-1569_1992_029_0405_asciso_2.3.co_2

[11] Mølsted K Asher-McDade C Brattström V A six-center international study of treatment outcome in patients with clefts of the lip and palate: part 2. Craniofacial form and soft tissue profile. Cleft Palate Craniofac J. 1992;29:398–404. 1472516 10.1597/1545-1569_1992_029_0398_asciso_2.3.co_2

[12] Shaw WC Asher-McDade C Brattström V A six-center international study of treatment outcome in patients with clefts of the lip and palate: part 1. Principles and study design. Cleft Palate Craniofac J. 1992;29:393–397. 1472515 10.1597/1545-1569_1992_029_0393_asciso_2.3.co_2

[13] Shaw WC Dahl E Asher-McDade C A six-center international study of treatment outcome in patients with clefts of the lip and palate: part 5. General discussion and conclusions. Cleft Palate Craniofac J. 1992;29:413–418. 1472519 10.1597/1545-1569_1992_029_0413_asciso_2.3.co_2

[14] Long RE Jr Hathaway R Daskalogiannakis J The Americleft study: an inter-center study of treatment outcomes for patients with unilateral cleft lip and palate: part 1. Principles and study design. Cleft Palate Craniofac J. 2011;48:239–243. 21219224 10.1597/09-180.1

[15] Sell D Issues in perceptual speech analysis in cleft palate and related disorders: a review. Int J Lang Commun Disord. 2005;40:103–121. 16101269 10.1080/13682820400016522

[16] Lohmander A Willadsen E Persson C Methodology for speech assessment in the Scandcleft project–an international randomized clinical trial on palatal surgery: experiences from a pilot study. Cleft Palate Craniofac J. 2009;46:347–362. 19642772 10.1597/08-039.1

[17] Henningsson G Kuehn DP Sell D; Speech Parameters Group. Universal parameters for reporting speech outcomes in individuals with cleft palate. Cleft Palate Craniofac J. 2008;45:1–17. 18215095 10.1597/06-086.1

[18] John A Sell D Sweeney T The cleft audit protocol for speech-augmented: a validated and reliable measure for auditing cleft speech. Cleft Palate Craniofac J. 2006;43:272–288. 16681400 10.1597/04-141.1

[19] Sell D John A Harding-Bell A Cleft audit protocol for speech (CAPS-A): a comprehensive training package for speech analysis. Int J Lang Commun Disord. 2009;44:529–548. 18821108 10.1080/13682820802196815

[20] Chapman KL Baylis A Trost-Cardamone J The Americleft speech project: a training and reliability study. Cleft Palate Craniofac J. 2016;53:93–108. 25531738 10.1597/14-027 PMC5693235

